# Treatment outcome, treatment retention, and their predictors among clients of five outpatient alcohol treatment centres in Switzerland

**DOI:** 10.1186/s12889-016-3294-4

**Published:** 2016-07-16

**Authors:** Severin Haug, Michael P. Schaub

**Affiliations:** Swiss Research Institute for Public Health and Addiction, Zurich University, Konradstrasse 32, CH- 8031 Zurich, Switzerland

**Keywords:** Alcohol, Treatment outcome, Treatment retention, Predictors

## Abstract

**Background:**

Few studies have reported on the outcomes of outpatient alcohol treatment or the factors associated with effective treatment. We investigated treatment outcome, treatment retention, and their predictors in clients receiving outpatient treatment for alcohol misuse.

**Methods:**

Naturalistic, longitudinal multi-centre study in Switzerland that included 858 clients receiving outpatient treatment for alcohol misuse. Assessments were conducted at treatment admission, discharge, and 6- and 12-month follow-ups. Non-problem drinking was used as an indicator of positive treatment outcome.

**Results:**

Clients admitted to outpatient alcohol treatment were highly heterogeneous in terms of pre-treatment alcohol use and drinking goals. 45 % of clients exhibiting problem drinking at the beginning of treatment showed non-problem drinking at discharge, and 41 % and 43 % showed non-problem drinking at the 6- and 12-month follow-up, respectively; 51 % were discharged regularly and 43 % were discharged irregularly. Non-problem drinking at the 12-month follow-up was more likely in clients with a higher life satisfaction, those with lower alcohol use, those aiming for alcohol abstinence, and those who had been admitted for the first time to a treatment institution, whereas it was less likely in clients with a higher educational level. Treatment retention was higher among older clients, clients with a higher life satisfaction, and clients who subsisted on their own income.

**Conclusion:**

Irregular discharge is high in outpatient alcohol treatment; nevertheless, a substantial portion of clients can achieve and maintain non-problem drinking by a 12-month follow-up.

## Background

Only a minority of individuals suffering from alcohol use disorders seek professional help [1]. Nevertheless, increasing the availability of effective treatment interventions for such disorders could reduce alcohol-attributable mortality [2, 3]. Lower-threshold treatment options along with more individualized and diversified treatments could contribute to this objective [4].

Compared to inpatient treatment, outpatient treatment is a lower-threshold and lower-cost alternative [5]. Although a substantial number of people suffering from alcohol use disorders receive outpatient treatment [6], there have been a few studies on the efficacy of outpatient alcohol treatment or the factors associated with effective treatment. The majority of studies on outpatient treatment focused on patients with alcohol dependence and who were receiving abstinence-oriented treatment [5, 7–14]. These studies showed that outpatient treatment has relatively long-lasting positive effects, with around half of all patients studied being abstinent at a two- to five-year follow-up [5, 7, 12].

The baseline predictors of relapse identified in previous studies on outpatient alcohol treatment were prior detoxification [10], a greater number of alcohol-related problems and years of heavy alcohol consumption [14], earlier onset of alcohol dependence [9], higher severity of alcohol dependence, a greater number of prior treatments, higher levels of depression and anxiety [13], suicidal attempts prior to treatment [9], and a fewer number of positive life events before treatment [5]. Furthermore, various demographic predictors of relapse have been identified, including lower socio-economic level [14], lower secondary school qualification, no professional training [9], and younger age [7]. The results concerning gender remain controversial, with several studies revealing that female gender is associated with relapse [5, 14] and others indicating that it is associated with achievement of abstinence [7].

Regarding treatment characteristics, treatment dropout has been found to significantly predict relapse [5, 10]; thus, it represents a major barrier to successful treatment outcomes. A systematic review [15] of dropout from addiction treatment revealed that it was particularly high within non-residential and outpatient treatment settings. Across all 122 studies included in this review, cognitive deficits, low treatment alliance, personality disorders, and younger age were the most consistent risk factors of dropout [15]. However, to date, only one study examined the factors associated with retention in outpatient alcohol treatment. This study, conducted in Brazil, revealed that the use of adjuvant medication, severe alcohol dependence, higher age, and higher frequency of alcohol consumption were associated with better treatment retention after four weeks of treatment [11].

Although intensive abstinence-oriented treatment for clients with severe alcohol use disorders still represents a substantial proportion of outpatient treatment programs, these programs are nowadays characterized by a considerable diversity of treatment modalities (eg considering controlled drinking goals) and treatment durations (eg including short-term treatment) [16, 17]. In Switzerland, almost all (90 %) outpatient alcohol treatment services provide information on controlled drinking as a treatment objective and around 81 % and 64 % offer controlled drinking as the final objective for alcohol misusers and patients with alcohol dependency, respectively [16]. This underlines the need for studies with broader inclusion and outcome criteria.

Considering this, we conducted a multi-centre, longitudinal, naturalistic effectiveness study, that is, a study carried out under the conditions of routine counselling practice, including clients with heterogeneous problem severity and comorbid disorders, as well as counsellors who apply exactly those methods that they usually do and are experienced in [18]. This study, conducted in Switzerland, included assessments at treatment admission, treatment discharge, and at 6- and 12-month follow-ups and investigated outcomes of alcohol treatment as well as the predictors of treatment retention and outcome. Because clients of outpatient treatment are highly heterogeneous in terms of alcohol use at admission, and for a substantial portion of clients the outpatient treatment was aftercare following inpatient treatment or alcohol detoxification, we examined treatment outcomes separately for clients with problem drinking at baseline and those without.

## Methods

### Study design and main outcome

This longitudinal, multi-centre naturalistic study on the effectiveness of outpatient alcohol treatment in Switzerland was conducted between March 2011 and January 2015. Because of the heterogeneity of clients in outpatient alcohol treatment in terms of drinking goals and alcohol use, and in contrast to previous studies in outpatient settings, we did not use alcohol abstinence as an indicator of positive treatment outcome. Instead, we defined a positive treatment outcome as non-problem drinking according to the consumption items of the Alcohol Use Disorders Identification Test (AUDIT-C) [19]. Treatment retention was defined as regular discharge with or without transition to another institution (see also the section on measures and instruments). Assessments were conducted at treatment admission and treatment discharge, and at 6- and 12- month follow-ups. This study was approved by the Local Ethics Committee of the Canton of Zurich, Switzerland (KEK-StV-Nr. 05/11). All study participants gave their written informed consent.

Four of the participating treatment centres initiated this study and one centre joined the study after the study procedures had been defined. The study authors, who were from the external Swiss Research Institute for Public Health and Addiction, were responsible for the study design, outcome measures, data analysis, and publications.

### Participants

Study participants were recruited from 5 Swiss outpatient alcohol treatment centres in the greater areas of Berne (Stiftung Berner Gesundheit and Blaues Kreuz Bern), Zurich (Zürcher Fachstelle für Alkoholprobleme), Aarau (Aargauische Stiftung Suchthilfe), and Baden (Beratungszentrum Bezirk Baden). Although the participating centres provide treatment for clients in various regions of Switzerland, both residential (rural/urban) and language (French and German), the treatment centres and the data collected are not representative of all outpatient alcohol treatment centres in Switzerland.

Clients who had entered treatment between March 2011 and November 2012 and who had completed treatment before December 2013 were invited to participate if they met the following inclusion criteria: (1) their own alcohol consumption was the main reason for treatment and (2) at least 3 counselling sessions were provided during treatment. Clients were excluded from study participation if they met one or more of the following criteria: (1) they had cognitive impairments or language difficulties that did not allow them to complete the questionnaires, (2) they were being represented by a legal guardian, or (3) they had had an acute emergency situation.

Within the study period, a total of 2,513 clients entered treatment due to their own alcohol consumption. Of these, 340 (13.5 %) were excluded because of one or more of the above mentioned exclusion criteria. A further 925 (36.8 %) were excluded for having less than 3 counselling sessions throughout treatment. Thus, a total of 1,248 persons were eligible for study participation. Of these, 1,009 (80.8 %) provided informed consent. The 858 clients who finished treatment before December 2013 represented the sample for analysis within the present study. Out of the 858 clients participating in the study, 311 (36.2 %) completed measures at the end of treatment, 532 (62.0 %) at the 6-month follow-up, and 512 (59.7 %) at the 12-month follow-up.

### Treatment content

We conducted no systematic assessment of treatment content. However, all of the involved institutions utilized motivational interviewing approaches (ie the pros and cons of alcohol abstinence and alcohol reduction; strategies for goal achievements) [20], the principles of cognitive behavioural therapy (identifying risk situations, situational analysis, relapse prevention), and behavioural self-management (drinking diary).

### Measures and instruments

The following data were assessed at treatment admission by the counsellor as part of the routinely applied information network on addiction care and therapy in Switzerland (ie ‘act-info’): (1) sex, (2) age in years (continuous), (3) nationality, (4) education level, (5) means of subsistence, (6) partnership status, (7) whether there are children living in the household, (8) aftercare following alcohol withdrawal treatment, (9) referring person or institution, and (10) whether this was the first or the second or further admission to the respective alcohol treatment centre.

The type of treatment completion was also assessed by the counsellor using the following response options: (1) regular discharge without transition to another institution, (2) regular discharge with transition to another institution, (3) change of residence, (4) hospitalisation, (5) imprisonment, (6) loss of contact, (7) discontinuation of treatment, and (8) death. Participants were assigned to the treatment retention group (or ‘regular treatment discharge’ group) if their counsellor selected response options (1) or (2). Response options (3)–(8) were considered examples of irregular discharge.

The following data were assessed via self-administered questionnaire (completed by clients) at the beginning and end of treatment as well as at the 6- and 12-month follow-ups: (1) alcohol use, (2) general health status, (3) life satisfaction, and (4) drinking goal.

Alcohol use was assessed using the short form of the Alcohol Use Disorders Identification Test, the AUDIT-C [19]. This comprises 3 items on (1) frequency of alcohol consumption (‘How often do you have a drink containing alcohol?’ with the response options ‘never’, ‘monthly or less’, ‘2–4 times a month’, ‘2–3 times a week’, and ‘4 or more times a week’), (2) quantity of alcohol consumption (‘How many drinks do you have on a typical day when you are drinking?’ with the response options ‘1–2 drinks’, ‘3–4 drinks’, ‘5–6 drinks’, ‘7–9 drinks’, and ‘10 or more drinks’; pictures were used to illustrate the quantity of a standard drink, which corresponded to 12–14 g of pure alcohol), and (3) binge drinking (‘How often do you have 6 or more drinks on one occasion?’ with the response options ‘never’, ‘less than monthly’, ‘monthly’, ‘weekly’, and ‘daily or almost daily’). Compared to other screening questionnaires, the AUDIT-C showed good psychometric properties and has clear advantages because of its brevity [21]. Based on a recent validation study of a large German sample, a cut-off of ≥4 for women and ≥5 for men was used to define problem drinking [22].

Self-rated general health [23] was assessed via the item ‘Would you say your health in general is: (1) excellent, (2) very good, (3) good, (4) fair, or (5) poor?’ Life satisfaction was assessed by using the Questions on Life Satisfaction instrument [24], which covers eight areas of life usually relevant to some degree to everyone in the Western world: friends/acquaintances, leisure time/hobbies, health, income/financial security, occupation/work, housing/living conditions, family life/children, and partner relationship/sexuality. The participants rated their satisfaction with each area on a 5-point scale ranging from ‘not satisfied’ to ‘very satisfied’. The total score, which is the sum of the eight item scores, ranges from 8–40. A psychometric evaluation of the Questions on Life Satisfaction demonstrated that this instrument has a high level of internal consistency and adequate sensitivity and construct validity [24].

Drinking goals were assessed by the item ‘Which is currently your personal goal concerning alcohol consumption?’ The response options were as follows: (1) ‘I want to be abstinent’, (2) ‘I only want to drink a certain quantity of alcohol’, (3) ‘I have not decided yet’, and (4) ‘I do not want to restrict myself’.

### Statistical analysis

First, we described the demographic, health-related, and treatment characteristics of clients at baseline. Second, we conducted non-response analyses (t-tests for continuous and *χ*^2^-tests for categorical variables) to examine whether the study participants who completed the questionnaires at treatment discharge and the follow-up assessments differed from those who did not respond to these assessments.

Third, we determined the number and percentages of study participants who exhibited a positive treatment outcome at discharge and at the 6- and 12-month follow-ups. As noted before, a positive treatment outcome was defined as non-problem drinking according to the AUDIT-C [19] using the cut-offs of ≥4 for women and ≥5 for men [22]. Clients of outpatient treatment are highly heterogeneous in terms of alcohol use at admission, and for a substantial portion of clients, the outpatient treatment was aftercare following inpatient treatment or alcohol detoxification. Thus, we decided to examine treatment outcome separately for clients with problem drinking at baseline and those without. Treatment outcome was also determined separately (1) considering all available data at each time of measurement and (2) using an imputed dataset wherein missing values at discharge and follow-up assessments were replaced via the multiple imputation [25] procedure of SPSS [26]. The main advantages of multiple imputation are that it results in less biased estimates, providing more validity than complete case analyses or other approaches used to deal with missing data. Furthermore, it uses all available data, thereby preserving sample size and statistical power [27]. Particularly, significant differences between responders and non-responders within this study underlined the need to impute missing data both at treatment discharge and at the follow-up assessments. We created 20 imputed datasets using all available data (demographic-, health-, and alcohol- and treatment-related variables) at admission, discharge, and follow-up.

Finally, we performed separate binary logistic regression analyses (subsequently referred to as the ‘univariate analyses’) to evaluate the ability of each client characteristic to predict treatment retention and outcome at the 12-month follow-up. After examining the univariate predictors, a multivariate prediction model was developed for each dependent variable. As suggested by [28], variable selection comprised the following steps: (1) significant predictors (*p* < .05) from the univariate analyses were entered into the preliminary multivariate model; (2) variables that were non-significant at *p* > .05 were removed one at a time, with those with the highest p-values being removed first (backward selection); and (3) to account for suppressor effects, the resulting model was verified by adding each of the variables excluded in step (2) separately into the regression model. In this last step, only variables that remained significant at *p* < .05 were retained in the final model (forward selection). All data were analysed using SPSS Statistics 22. All statistical tests were two-tailed and had significance levels at *p* < .05.

## Results

### Client characteristics at admission

The characteristics of study participants at admission are displayed in Table 1. Of the 858 study participants, 563 (65.6 %) were male. The mean age of the whole sample was 45.3 years. Problem drinking (according to the AUDIT-C cut-offs mentioned previously) was found in 564 (65.7 %) participants.

**Table 1:** Client characteristics at treatment admission

	Study participants (*n* = 858)
Sex	
Female	295 (34.4 %)
Male	563 (65.6 %)
Age in years, *M (SD)*	45.3 (12.5)
18–30	121 (male: 93 [76.9 %])
31–50	427 (male: 279 [65.3 %])
51–84	298 (male: 186 [62.4 %])
*Missing*	*12 (1.4 %)*
Nationality	
Swiss	739 (86.1 %)
Other (mainly German, Italian, French, or Portuguese)	112 (13.1 %)
*Missing*	*7 (0.8 %)*
Educational level	
Low (no or partly completed compulsory education)	114 (13.3 %)
Medium (compulsory or vocational education/apprenticeship)	430 (50.1 %)
High (higher vocational education or university)	149 (17.4 %)
*Missing*	*165 (19.2 %)*
Means of subsistence	
Own income	471 (54.9 %)
Savings or pension	131 (15.3 %)
Social welfare	172 (20.0 %)
Partner or family members	65 (7.6 %)
Other	12 (1.4 %)
*Missing*	*7 (0.8 %)*
Partnership status	
No or temporary partnership	333 (38.8 %)
Stable, living apart	103 (12.0 %)
Stable, living together	362 (42.2 %)
*Missing*	*60 (7.0 %)*
Children living in the household	
No	654 (76.2 %)
Yes	196 (22.8 %)
*Missing*	*8 (0.9 %)*
Self-rated general health	
Excellent/very good	255 (29.7 %)
Good	384 (44.8 %)
Poor	190 (22.1 %)
*Missing*	*29 (3.4 %)*
Life satisfaction (Questions on Life Satisfaction, scores ranging from 10–40), *M (SD)*	27.6 (6.2)
*Missing*	*27 (3.1 %)*
Problem drinking (AUDIT-C cut-offs)	
Yes	564 (65.7 %) (male: 356 [63.2 %], female: 208 [70.5 %])
No	260 (30.3 %) (male: 187 [33.2 %], female: 73 [24.7 %])
*Missing*	*34 (4.0 %)*
Drinking goal	
I do not want to restrict myself	8 (0.9 %)
Controlled drinking	375 (43.7 %)
Abstinence	350 (40.8 %)
Have not yet decided	72 (8.4 %)
*Missing*	*53 (6.2 %)*
Admission to respective alcohol treatment centre	
First admission	611 (71.2 %)
Second or further admission	247 (28.8 %)
Treatment assignment	
Own initiative	312 (36.4 %)
Partner, family, or friends	100 (11.7 %)
Health institution	246 (28.7 %)
Social services	43 (5.0 %)
Judge	99 (11.5 %)
Employer or teacher	32 (3.7 %)
*Missing*	*26 (3.0 %)*
Aftercare following alcohol withdrawal treatment	
No	708 (82.5 %)
Yes	135 (15.7 %)
*Missing*	*15 (1.7 %)*

*Notes:* Values are numbers (%) unless specified otherwise. *AUDIT-C* consumption items of the alcohol use disorders identification test. AUDIT-C cut-offs for problem drinking were ≥5 and ≥4 for men and women, respectively

### Treatment characteristics and treatment discharge

The mean duration of treatment was 225.8 days (*SD* = 185.9) with a mean of 9.7 (*SD* = 7.9) individual and 0.8 (*SD* = 3.3) group sessions provided. A total of 433 (50.5 %) participants were discharged regularly with (*n* = 31; 3.6 %) or without (*n* = 402; 46.9 %) transition to another institution. In contrast, 366 (42.7 %) participants were discharged irregularly because of a change of residence (*n* = 11; 1.3 %), hospitalisation (*n* = 4; 0.5 %), imprisonment (*n* = 3; 0.3 %), loss of contact (*n* = 303; 35.3 %), discontinuation of treatment (*n* = 40; 4.7 %), or death (*n* = 5; 0.6 %). Data concerning discharge were missing for 59 participants (6.9 %).

### Non-response analysis

In comparing participants who completed the questionnaires at treatment discharge and those who did not, we found that non-responders had a poorer health status (*χ*^2^ = 12.7, *p* < .01) and lower life satisfaction (t = 5.4, *p* < .01); were less often assigned to treatment by a judge, employer, or teacher (*χ*^2^ = 23.4, *p* < .01); more often subsisted on monetary resources other than their own income (*χ*^2^ = 14.7, *p* < .01); and were more often discharged irregularly (*χ*^2^ = 285.2, *p* < .01).

At the 6-month follow-up, non-responders were younger than were responders (t = 2.7, *p* = .01); furthermore, they more often subsisted on monetary resources other than their own income (*χ*^2^ = 7.9, *p* = .05), attended fewer group sessions (t = 2.2, *p* = .03), and were more often discharged irregularly (*χ*^2^ = 36.8, *p* < .01).

Non-responders at the 12-month follow-up were also significantly younger than were responders (t = 5.1, *p* < .01). Furthermore, they more often did not have Swiss nationality (*χ*^2^ = 8.4, *p* < .01), subsisted on monetary resources other than their own income (*χ*^2^ = 13.2, *p* < .01), tended to have a lower life satisfaction (t = 3.0, *p* < .01), and were more often discharged irregularly (*χ*^2^ = 40.9, *p* < .01).

### Treatment outcome

Due to selective non-response, eg of clients with irregular discharge, the percentage of clients with a positive treatment outcome (ie non-problem drinking) was higher when we considered only the available data compared to the imputed data (Table 2). Using the imputed data, 45.2 % of clients with problem drinking at admission showed non-problem drinking at the end of treatment, and 41.1 % and 43.2 % showed non-problem drinking at the 6- and 12-month follow-ups, respectively. Among clients with non-problem drinking at admission, some of whom were receiving outpatient treatment as aftercare following inpatient detoxification, 86.6 % remained non-problem drinkers at the end of treatment, while 79.6 % and 80.4 % showed non-problem drinking at the 6- and 12-month follow-ups, respectively.

**Table 2:** Percentage of clients with a positive treatment outcome (non-problem drinking according to the AUDIT-C) at discharge and follow up assessments, separated by problem drinking at treatment admission

	Discharge		6-months follow up		12-months follow up	
	Available data (*n* = 308) (95 % CI)	Imputed data	Available data (*n* = 523) (95 % CI)	Imputed data	Available data (*n* = 502) (95 % CI)	Imputed data
Problem drinking at admission	55.5 % (48.7–62.8 %)	45.2 %	44.4 % (39.0–49.6 %)	41.1 %	46.1 % (40.7–51.5 %)	43.2 %
Non-problem drinking at admission	93.1 % (88.2–98.0 %)	86.6 %	81.9 % (75.5–87.7 %)	79.6 %	83.0 % (76.5–88.9 %)	80.4 %

*Notes:* Available data: all available data at any time of assessment are considered; Imputed data: missing values were imputed using multiple imputation

*AUDIT-C* consumption items of the alcohol use disorders identification test, *95 % CI* 95 % confidence interval. AUDIT-C cut-offs for problem drinking were ≥5 and ≥4 for men and women, respectively

### Predictors of outcome at 12-month follow-up

The univariate predictors of a positive treatment outcome at the 12-month follow-up are presented in Table 3. The results of the multivariate regression model predicting non-problem drinking at the 12-month follow-up are displayed in Table 4. We found a greater likelihood of a positive treatment outcome among clients with (1) a lower or medium educational level, (2) a higher life satisfaction, (3) non-problem drinking at admission, (4) a goal of abstinence compared to controlled drinking, and (5) first-time admission to a treatment institution.

**Table 3:** Univariate predictors of a positive treatment outcome (ie non-problem drinking according to the AUDIT-C cut-offs) at 12-month follow-up and treatment retention

Client characteristic at treatment admission	Non-problem drinking at 12-month follow-up OR (95 % CI)	Treatment retention OR (95 % CI)
Sex		
Male *(Ref.)*		
Female	0.93 (0.64–1.35)	0.83 (0.61–1.11)
Age in years	1.02 (1.01–1.04)**	1.02 (1.00–1.03)**
Nationality		
Swiss *(Ref.)*		
Other	1.69 (0.91–3.14)	0.91 (0.60–1.38)
Educational level		
Low *(Ref.)*		
Medium	1.20 (0.68–2.12)	1.01 (0.65–1.57)
High	0.47 (0.24–0.91)*	1.29 (0.77–2.18)
Means of subsistence		
Own income *(Ref.)*		
Savings or pension	0.68 (0.42–1.13)	0.62 (0.41–0.92)*
Social welfare	1.24 (0.75–2.06)	0.43 (0.30–0.63)**
Partner or family members	0.71 (0.35–1.43)	0.92 (0.53–1.59)
Partnership status		
No or temporary partnership *(Ref.)*		
Stable, living apart	1.34 (0.71–2.56)	0.95 (0.59–1.51)
Stable, living together	1.29 (0.88–1.91)	1.63 (1.19–2.23)**
Children living in the household		
No *(Ref.)*		
Yes	1.01 (0.62–1.67)	1.09 (0.71–1.65)
Self-rated general health		
Excellent/very good *(Ref.)*		
Good	0.58 (0.38–0.89)*	0.58 (0.42–0.81)**
Poor	0.67 (0.41–1.10)	0.52 (0.35–0.77)**
Life satisfaction (score 10–40)	1.07 (1.04–1.11)**	1.09 (1.07–1.12)**
Problem drinking according to AUDIT-C cut-offs		
No *(Ref.)*		
Yes	0.18 (0.11–0.28)**	0.66 (0.48–0.90)**
Drinking goal		
Abstinence *(Ref.)*		
I do not want to restrict myself	0.43 (0.09–1.98)	2.50 (0.50–12.58)
Controlled drinking	0.23 (0.16–0.35)**	1.04 (0.76–1.40)
Have not yet decided	0.35 (0.18–0.70)**	0.69 (0.41–1.18)
Admission to respective alcohol treatment centre		
First admission *(Ref.)*		
Second or further admission	0.58 (0.40-0.86)**	0.71 (0.52–0.97)*
Treatment assignment		
Own initiative *(Ref.)*		
Partner, family, or friends	2.46 (1.37–4.44)**	0.88 (0.55–1.41)
Health institution	2.08 (1.34–3.24)**	1.00 (0.71–1.43)
Social service	1.23 (0.47–3.25)	0.49 (0.25–0.98)*
Judge	4.07 (2.05–8.09)**	2.55 (1.50–4.32)**
Employer or teacher	3.29 (1.23–8.77)*	1.85 (0.81–4.22)
Aftercare following alcohol withdrawal treatment		
No *(Ref.)*		
Yes	1.58 (0.94–2.64)	0.65 (0.44–0.96)*

AUDIT-C cut-offs for problem drinking were ≥5 and ≥4 for men and women, respectively

*Notes: AUDIT-C* consumption items of the alcohol use disorders identification test, *OR* odds ratio, *95 % CI* 95 % confidence interval, *Ref*. reference category

*p < .05; **p < .01

**Table 4:** Final multivariate regression model of client characteristics predicting positive treatment outcome (ie non-problem drinking according to the AUDIT-C cut-offs) at 12-month follow-up

Client characteristic at treatment admission	OR (95 % CI)
Educational level	
Low *(Ref.)*	
Medium	1.18 (0.60–2.31)
High	0.38 (0.17–0.83)*
Life satisfaction (Questions on Life Satisfaction)	1.06 (1.02–1.11)**
Problem drinking (AUDIT-C)	
No *(Ref.)*	
Yes	0.38 (0.21–0.67)**
Drinking goal	
Abstinence *(Ref.)*	
Controlled drinking	0.22 (0.13–0.37)**
I do not want to restrict myself	0.44 (0.04–4.74)
Have not yet decided	0.48 (0.21–1.07)
Admission	
First admission *(Ref.)*	
Readmission	0.43 (0.25–0.74)**

AUDIT-C cut-offs for problem drinking were ≥5 and ≥4 for men and women, respectively

*Notes: AUDIT-C* consumption items of the alcohol use disorders identification test, *OR* odds ratio, *95 % CI* 95 % confidence interval, *Ref*. reference category

*p < .05; **p < .01; Nagelkerke’s R^2^ = .30; *n* = 376

After controlling for client characteristics at treatment admission, we found that treatment retention was a significant predictor of a positive treatment outcome (OR 1.95, 95 % CI 1.33-2.85, *p* < .01), with 64.5 % of clients with regular discharge and 48.2 % of those with irregular discharge showing non-problem drinking at the 12-month follow-up.

### Predictors of treatment retention

The univariate predictors of treatment retention are presented in Table 3. The results of the multivariate regression model predicting treatment retention are displayed in Table 5. Ultimately, we found a higher likelihood of treatment retention in (1) older clients and (2) clients with a higher life satisfaction. However, we found a lower likelihood of retention among clients who subsisted on their savings, pension, or social welfare compared to those who subsisted on their own income.

**Table 5:** Final multivariate regression model of client characteristics predicting regular treatment discharge

Client characteristic at treatment admission	OR (95 % CI)
Age in years	1.02 (1.01–1.03)**
Means of subsistence	
Own income *(Ref.)*	
Savings or pension	0.48 (0.30–0.77)**
Social welfare	0.61 (0.41–0.91)*
Partner or family members	1.10 (0.62–1.96)
Life satisfaction (Questions on Life Satisfaction)	1.08 (1.05–1.11)**

*Notes: OR* odds ratio, *95 % CI* 95 % confidence interval, *Ref* reference category

*p < .05; **p < .01; Nagelkerke’s R^2^ = .12; *n* = 742

## Discussion

We investigated treatment outcome and the predictors of treatment retention and outcome in clients receiving outpatient alcohol treatment in Switzerland. While previous studies on this topic focused exclusively on patients with present alcohol use disorders, clients in our study were more heterogeneous, as we included clients without alcohol use due to prior detoxification at another institution and those who reduced their alcohol use prior to treatment admission to a non-hazardous level. There were four main findings: (1) Clients admitted to outpatient alcohol treatment were highly heterogeneous in terms of pre-treatment alcohol use and drinking goals. (2) Approximately 4 out of 10 clients with problem drinking at the beginning of treatment showed non-problem drinking at the 6- and 12-month follow-up assessments; in contrast, approximately 8 out of 10 initially non-problem drinking clients, some of whom were receiving outpatient treatment as aftercare following detoxification, remained non-problem drinkers at the 6- and 12-month follow-up assessments. (3) Positive treatment outcome was more likely in clients with a higher life satisfaction, those with lower alcohol use, those aiming for alcohol abstinence, and those who had been admitted into a treatment institution for the first time; in contrast, the likelihood of a positive outcome was lower among clients with a higher educational level. (4) Treatment retention was higher among older clients, clients with a higher life satisfaction, and clients who subsisted on their own income.

The heterogeneity of participants concerning alcohol use and drinking goals reflects the naturalistic study design, as we did not exclude clients without present alcohol use disorders and there is a relatively large distribution of outpatient treatment programs focusing on controlled drinking in Switzerland [16]. However, this heterogeneity also precludes comparison of treatment outcome results with those of previous studies, which typically focused on abstinence-oriented treatment in clients presenting alcohol use disorders [5, 9, 12, 13]. The results of the present study indicate that for problem drinkers at admission, most of the achievements made during outpatient alcohol treatment were maintained over the study period; we observed only slightly worse treatment outcomes at the 6- and 12-month follow-ups compared to at treatment discharge. These results will help providers of similar outpatient alcohol treatment programs by providing figures for comparison, and further allow for an estimation of the probability that clients with and without initial problem drinking will achieve and maintain a positive treatment outcome.

Although we cannot directly compare our results concerning treatment outcome with previous studies, we can still do so with the predictors of treatment outcome. These findings were generally in line with previous studies. Similar to findings on abstinence-oriented outpatient alcohol treatments [5, 10], our study showed that treatment dropout or irregular discharge was negatively associated with treatment outcome. Furthermore, similar to previous studies on outpatient alcohol treatment [5, 13, 14], greater alcohol use, previous treatments, and higher levels of depressive symptoms and anxiety, which are associated with lower life satisfaction, were predictors of a negative treatment outcome.

In line with our results, previous studies that assessed the impact of self-selected drinking goals on treatment outcome (eg [29–31] revealed better outcomes among those who chose abstinence compared to controlled drinking. However, differences in several characteristics that we did not account for, eg in the motivation to change, between goal abstainers and those aiming for controlled drinking should be considered when interpreting this result [29].

The finding that higher treatment retention is more likely in older clients was in line with a previous study from Brazil [11]; however, unlike our study, this previous study revealed that severity of alcohol dependence and greater frequency of alcohol consumption were also associated with better treatment retention [15]. In our study, higher life satisfaction, an indicator of lower illness severity, was positively associated with treatment retention.

Several limitations of this study must be mentioned. First, this was a naturalistic longitudinal study that lacked a control group. Therefore, our study cannot provide sufficient evidence of the efficacy of outpatient alcohol treatment; rather, it can only provide estimates of the rate of successful outcome. Second, due to temporal restrictions of study duration, the study did not address longer-term outcomes beyond 12 months. Third, also in connection with the naturalistic study design, we could not assess all of the potential predictors of treatment outcome and retention derived from previous studies. Fourth, outcome data on alcohol use were self-reported and not biochemically verified or cross-validated with other data. Fifth, we did not carry out a systematic assessment of the content for each of the outpatient treatment programmes delivered.

Some of our results might have implications for the provision of outpatient alcohol treatment. First, considering the higher dropout rates among younger clients, those with a lower life satisfaction, and those subsisting on their savings, pension, or social welfare, counsellors might have to make a particular effort to keep these subgroups in treatment, such as by maintaining a good therapeutic relationship. Furthermore, measures such as proactive phone calls in the case of non-compliant individuals or appointment reminders sent via text message might help to increase treatment retention [32]. Considering the better treatment outcome among clients aiming at abstinence compared to those aiming at controlled drinking, clients who are uncertain which goal to pursue should be advised to abstain.

## Conclusions

In conclusion, this study shows that clients receiving outpatient alcohol treatment in Switzerland are very heterogeneous in terms of their pre-treatment alcohol use and drinking goals. Studies on the efficacy of outpatient alcohol treatment should consider this heterogeneity by adopting broad inclusion and outcome criteria and presenting results for different subgroups, eg clients differing in terms of initial alcohol use or drinking goal. The study results on treatment outcome show that a substantial portion of clients can achieve and maintain non-problem drinking until a 12-month follow-up. However, to obtain better estimates of the efficacy of outpatient alcohol treatment, future controlled studies are required, with a random assignment of clients to treatment vs. no-treatment or different treatment options. Given the lower costs of outpatient compared to inpatient and day-hospital treatment [8], future studies should additionally integrate measures on treatment costs and cost-effectiveness of alcohol treatment in order to inform policy decisions.

## Abbreviations

95 % CI, 95 % confidence interval; AUDIT-C, consumption items of the alcohol use disorders identification test; OR, odds ratio; Ref., reference category
